# The Importance of Dental Treatment in Patients Before Radiotherapy, Chemotherapy, and Cardiac Surgeries: A Narrative Review

**DOI:** 10.3390/jcm14176330

**Published:** 2025-09-08

**Authors:** Seyedamirreza Mostafavi, Magdalena Wyszyńska, Małgorzata Skucha-Nowak

**Affiliations:** 1 Division of Medical Sciences in Zabrze, Doctoral’s School, Medical University of Silesia in Katowice, 15 Poniatowskiego Street, 40-055 Katowice, Poland; 2 Department of Dental Materials, Division of Medical Sciences in Zabrze, Medical University of Silesia in Katowice, 15 Poniatowskiego Street, 40-055 Katowice, Poland; magdalena.wyszynska@sum.edu.pl; 3 Department of Dental Propedeutics, Division of Medical Sciences in Zabrze, Medical University of Silesia in Katowice, 15 Poniatowskiego Street, 40-055 Katowice, Poland; mskucha-nowak@sum.edu.pl

**Keywords:** radiotherapy, chemotherapy, cardiac surgical procedures, dental care, oral manifestation, radiotherapy adverse effects, chemotherapy adverse effects

## Abstract

Oncological patients or the individuals scheduled for cardiovascular surgeries are at risk of both oral and systemic complications when existing dental pathologies are not addressed before these therapies. This narrative review explores the current literature on the role of pre-treatment dental care in reducing unfavorable outcomes and promoting treatment efficacy in medically compromised patients. The data show that early dental intervention, particularly prior to head and neck radiotherapy, considerably reduces the risk of osteoradionecrosis, rampant radiation caries, and xerostomia. Chemotherapy-associated mucositis, infections, and microbial imbalance are similarly worsened by untreated oral disease but may be managed through early sanitation and hygiene reinforcement. In cardiac patients, conditions such as apical periodontitis and periodontitis may lead to bacteremia, infective endocarditis, or prosthetic valve infections, highlighting the systemic relevance of oral health. Tooth extractions, restorative treatment of carious lesions, and control of active oral infections performed before systemic therapy can reduce complication rates, improve treatment continuity, and enhance patients’ quality of life. Integrating comprehensive dental assessment into routine pre-treatment planning allows early identification of oral health risks and supports a multidisciplinary approach that optimizes overall clinical outcomes.

## 1. Introduction

The oral cavity is the gateway to the digestive system, and the various microorganisms that inhabit it are crucial to preserving the delicate equilibrium between health and illness both locally and systemically. Within this microenvironment, a variety of microbes—including bacteria, fungi, protozoa, and viruses—coexist and interact with each other and with the host, shaping oral and systemic health outcomes [1].

Disruption of this microbial equilibrium contributes to the development of dental diseases. For instance, the demineralization process of enamel is linked to an overabundance of species that are typically found in low abundance, such as lactobacilli, bifidobacteria, and mutans streptococci [2,3]. In early stages, carious lesions can be reversed through preventive or minimally invasive interventions, but if untreated, they progress to cavitations that require more extensive restorative or surgical treatments [4]. The most common sources of oral inflammation include untreated caries, periodontal disease, and oral infections such as periapical abscesses, candidiasis, and herpes simplex virus reactivation. These conditions stimulate the production of immunological mediators such as prostaglandins, cytokines, and reactive oxygen species, which are essential for normal immune function. However, when these pathways are excessively or chronically activated, they can cause tissue damage and contribute to broader systemic complications [4,5]. While they are necessary for immune system, excessive or chronic activation of these pathways can lead to tissue damage and broader systemic complications. The presence of severe dental pathology, such as gangrenous decay, periapical abscesses, or chronic periodontitis, poses additional risks for medically compromised individuals. In oncological or major surgeries like cardiac, orthopedic, or transplant procedures, oral infections may result in transient or persistent bacteremia, raising the likelihood of complications such as infective endocarditis or implant-associated infections [6,7]. Consequently, pretreatment dental assessment and intervention are critical in patients scheduled for head and neck radiotherapy, as they allow identification and management of oral risk factors before therapy begins, thereby reducing the incidence of complications such as osteoradionecrosis (ORN), xerostomia, and radiation-induced caries [8,9]. Teeth with a poor prognosis should ideally be extracted before therapy, as surgical interventions during or after radiation carry significant risk. Chemotherapy presents its own oral challenges, including mucositis, secondary infections, and delayed healing due to immunosuppression. Preventive oral sanitation, avoidance of trauma, and timely dental care can meaningfully reduce these side effects [9]. Current guidelines by MASCC/ISOO and ESMO emphasize the importance of collaboration between oncologic and dental teams in optimizing patient care outcomes [10,11]. Advances such as cone-beam computed tomography (CBCT) enable accurate diagnosis and planning [12]. Additionally, bioactive restorative materials, such as bioactive composites, can promote remineralization of dental tissues and enhance the durability of restorations in teeth compromised by radiotherapy [13]. Furthermore, regenerative therapies (e.g., stem cells), probiotics and zinc supplementation for oral microbiota modulation, and low-level laser therapy (LLLT) are emerging strategies to enhance mucosal healing and reduce complications [9,12,13]. By integrating preventive and therapeutic dental approaches into medical protocols, healthcare providers can significantly reduce the risk of systemic complications, improve treatment continuity, and enhance patient quality of life.

This article aims to review current evidence on dental prophylaxis before oncology therapy and cardiac surgery, with a focus on dental caries management. It also explores adjunctive therapies, including fluoride, saliva substitutes, and PRF, in reducing treatment-related complications and improving oral outcomes.

## 2. Search Strategy and Study Selection

**Figure 1:**
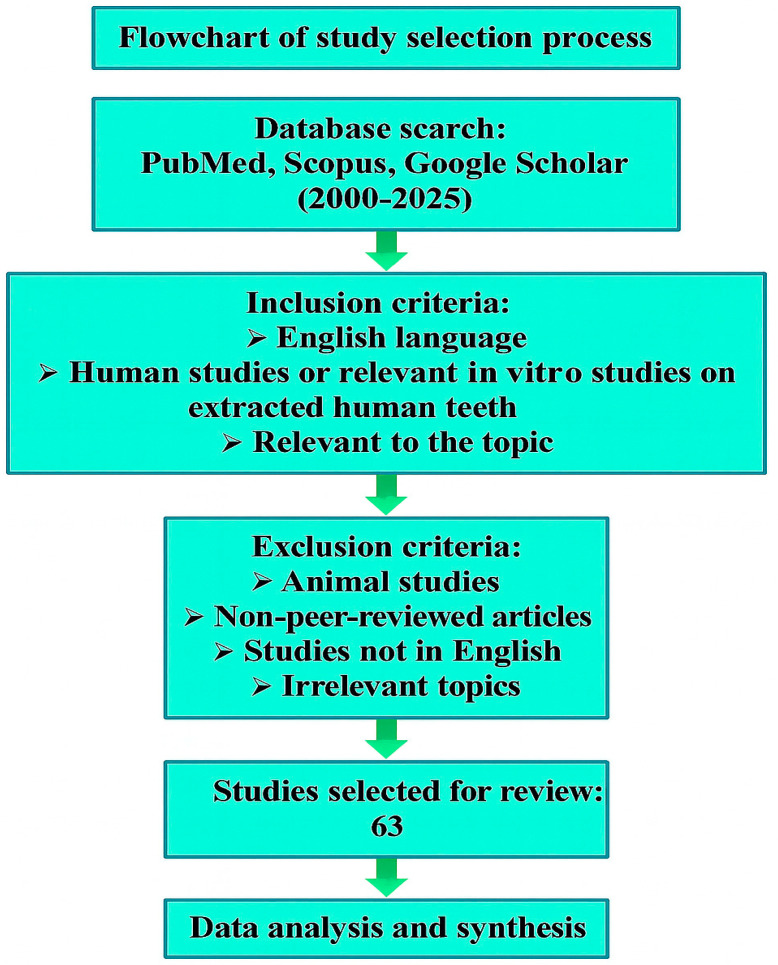
Flowchart of study selection process. Arrows indicate the sequential flow from database search through inclusion/exclusion criteria to final study selection.

A comprehensive literature search was conducted between 2000 and 2025 using the databases PubMed, Scopus, and Google Scholar. The search strategy combined the following keywords: (“dental treatment” OR “oral health” OR “dentistry”) AND (“radiotherapy” OR “chemotherapy” OR “oncology” OR “cancer treatment” OR “head and neck cancer” OR “cardiac surgery” OR “heart surgery”) AND (“pre-treatment” OR “before treatment” OR “preventive dentistry”). Articles were included if they were published in English; involved human participants or relevant in vitro studies on extracted human teeth; consisted of randomized controlled trials, observational studies, systematic reviews, narrative reviews, or meta-analyses; and were relevant to the topic. Studies not in English, animal studies, non-peer-reviewed articles, and irrelevant publications not related to pre-treatment dental care were excluded. Authors independently screened titles and abstracts, followed by a full-text assessment. Disagreements were resolved by consensus. A total of 63 studies met the inclusion criteria and were included in the review. The selection process is illustrated in Figure 1

## 3. Pathophysiology and Identification of Odontogenic Foci

Untreated odontogenic infections, including apical periodontitis, periodontal abscesses, and advanced carious lesions, can serve as sources of systemic infection [14].

Diagnosis of odontogenic foci requires a comprehensive clinical and radiographic evaluation. Clinicians should assess for signs such as tenderness to percussion, sinus tract formation, periodontal pocketing, and radiographic evidence of periapical radiolucency or alveolar bone loss. Cone beam computed tomography (CBCT) may provide enhanced visualization of periapical pathology or root fractures, improving diagnostic accuracy in complex cases [12,15].

## 4. Impact of Radiotherapy on Oral Health

The seventh most common cancer diagnosis globally is head and neck squamous cell carcinoma (HNSCC), a group of malignancies affecting the oral cavity, pharynx, hypopharynx, larynx, nasal cavity, and salivary glands [16].

One of the most frequently used methods to treat head and neck cancers (HNCs) is radiation therapy (RT), a method that utilizes ionizing radiation and semi-selectively destroys the genetic material of susceptible malignant cells, either directly or by generating free radicals that result in cell death. According to a study by Beech et al., RT causes adverse effects by destroying and damaging normal cells along with cancerous cells through the same mechanism of action mentioned earlier, particularly on those cells that are dividing rapidly [17]. Radiotherapy for head and neck cancers has progressed significantly over recent decades, particularly with the adoption of intensity-modulated radiation therapy (IMRT). This approach enables more accurate dose delivery while limiting exposure to surrounding healthy tissues. In the phase III multicenter PARSPORT trial, Nutting and colleagues found that parotid-sparing IMRT markedly reduced the incidence of moderate-to-severe xerostomia and improved both salivary function and quality of life compared with conventional techniques, with benefits persisting up to two years after treatment. Although the trial provided strong evidence for reduced toxicity, it was not designed to detect differences in survival or locoregional control. While these findings support the role of IMRT in mitigating certain complications, the long-term consequences for oral structures, including changes to the microbiota and jawbone, remain insufficiently understood [18]. Emerging modalities such as intensity-modulated proton therapy (IMPT) may offer further tissue sparing, although current clinical data on oral outcomes remain limited [19].

**Figure 2:**
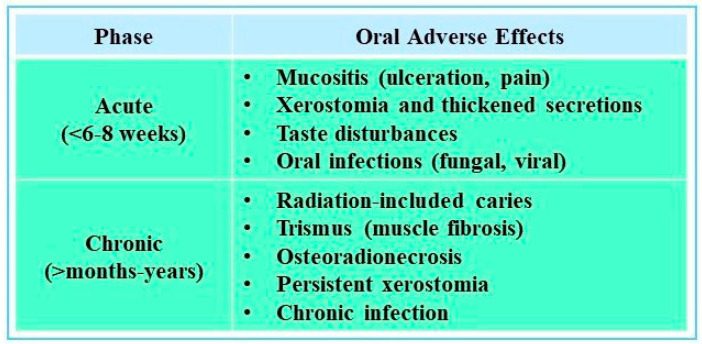
Acute vs. Chronic Oral Effects of Head and Neck Radiotherapy [7].

Regardless of the delivery method, radiation therapy can severely impact healthy oral tissues. The side effects can be due to complex microbiota, frequent and rapid replacement of oral epithelial cells, and damages to oral tissues during normal function [20]. Adverse effects of radiation on oral structures can be either acute or chronic, and they can occur directly or indirectly [21] (see Figure 2).

Acute complications of radiotherapy frequently include oral mucositis, which typically arises within the first two weeks of treatment and is characterized by erythema, ulceration, and pain that interfere with nutrition and medication adherence. Opportunistic fungal infections, most often candidiasis, may also develop, while progressive fibrosis of the masticatory muscles can lead to trismus. In the longer term, patients often experience taste disturbances, temporomandibular joint dysfunction, and osteoradionecrosis (ORN)—a serious complication of jawbone necrosis, particularly affecting the mandible—that significantly impair quality of life [9,10]. Treatment planning for head and neck malignancies often combines radiotherapy with surgery and, increasingly, chemotherapy. While effective, this multimodal approach increases the risk of oral and systemic side effects. Additionally, a cross-sectional study of 263 survivors revealed that at a median of 8.5 years after radiation therapy, 58% of the patients had xerostomia, 31% developed dysphagia, and 33% suffered from chronic fatigue [20]. Severity varies with patient age, radiation dose and field, and the presence of concurrent therapies [19,20]. These complications necessitate comprehensive dental management before, during, and after therapy to reduce morbidity and maintain treatment continuity [7].

### 4.1. Relationship Between Radiotherapy and Dental Caries

Radiotherapy-related damage to the salivary glands both reduce flow and alters salivary composition, disrupting key protective functions such as buffering, antimicrobial defense, and enamel remineralization. These changes directly contribute to the rapid development of radiation-induced caries, particularly affecting smooth and cervical surfaces. A prospective cohort study by Müller et al. confirmed measurable alterations in salivary composition and function after radiotherapy, though the small sample size limited the applicability of its findings [22]. More robust evidence is provided by the multicenter ORARAD study, which followed over 500 patients and demonstrated persistently reduced salivary flow and long-term xerostomia; both outcomes were strongly associated with an increased risk of radiation-induced caries [23].

Radiation-related caries (RRC) often manifests six to twelve months following the end of HNRT. RRC generally starts with minor fractures and cracks in the enamel and develops into a brown or blackish discoloration. RRC manifests as incisal/cuspal wear in conjunction with widespread cervical (amelocemental junction) caries, which can result in enamel delamination, dentin disintegration, and dental crown amputation. Compared to traditional caries, which primarily occur in pits, fissures, and the proximal portions of teeth, this is considered to be atypical pattern of clinical progression. If left untreated, RRC can progress rapidly and cause tooth structure loss, especially in areas under mechanical stress. Advanced decay may necessitate extractions, significantly increasing the risk of ORN [24,25]. In addition to indirect effects, radiation may directly alter the tooth structure, especially near the dentino–enamel junction, contributing to enamel delamination and tooth fragility. These structural changes, combined with oral environmental shifts, contribute to rampant, fast-progressing caries that threaten oral function and systemic health [7,25].

### 4.2. Importance of Pre-Radiotherapy Dental Assessment and Treatment

#### 4.2.1. Patient Education and Dietary Changes

Radiation-induced xerostomia frequently alters patients’ dietary behaviors, as many turn to soft, high-sugar foods and sweetened beverages to cope with oral dryness. Kawashima et al. (2024) demonstrated in an observational study that higher xerostomia severity was independently associated with more frequent intake of cariogenic foods, thereby increasing the risk of radiation-related dental caries [26]. This finding aligns with evidence from a recent systematic review by Alnaeem et al. (2025), which highlighted the consequences of xerostomia on nutrition, sleep quality, and overall quality of life in head and neck cancer patients [27]. These observations underscore the importance of integrating dietary counseling and structured patient education into pre- and post-radiotherapy care. Moreover, practical recommendations in clinical guidance stress the role of preventive strategies such as the use of sugar-free alternatives, frequent oral rinsing, and xylitol-containing products in mitigating caries risk and improving long-term outcomes in this vulnerable population [21].

#### 4.2.2. Fluoride and Remineralizing Agents

Historically, the daily use of 1% neutral sodium fluoride gel in custom trays was recommended to strengthen enamel and reduce caries risk. However, recent studies suggest the use of casein phosphopeptide–amorphous calcium phosphate (CPP-ACP), a stabilized source of bioavailable calcium and phosphate ions, which is a salivary biomimetic, has been shown to significantly reduce caries progression and enhance regression of early carious lesions in clinical studies on healthy individuals. When fluoride, in the form of sodium fluoride (NaF), was added to the CPP-ACP complex, increased remineralizing activity was seen. By creating a stannous fluorapatite barrier layer on the enamel surface, tin(II) fluoride (SnF_2_) has recently been proven to be more effective than NaF at preventing the breakdown of enamel minerals [28,29].

#### 4.2.3. The Choice of Dental Restorative Material

In patients receiving head and neck radiotherapy, the choice of restorative material is critical due to the elevated risk of radiation-related caries (RRC) and restoration failure [17]. De Amorim et al. (2021) conducted an in vitro study on extracted human teeth showing that ionizing radiation increases surface roughness and decreases microhardness of restorative materials, thereby potentially compromising their clinical performance and long-term durability [30]. In a more recent laboratory study, Atalay and Yazici (2024) similarly found that most bioactive restorative materials were adversely affected by radiotherapy [31]. Notably, resin composites and resin-modified glass ionomer cements (RMGICs) demonstrated comparatively greater resistance to radiation-induced degradation in surface properties [30,31].

#### 4.2.4. Surgical Considerations Pre-RT

Post-radiotherapy extractions are avoided due to the elevated risk of ORN; thus, any questionable teeth should be removed prior to therapy. Ideally, at least 7–14 days of healing should be allowed between extraction or other surgical procedures before the staring of radiotherapy [32]. In cases where bone healing is a concern, hyperbaric oxygen therapy (HBOT) may be considered to promote vascularization and bone recovery [25,32]. Some studies report improved post-extraction healing with platelet-rich fibrin; however, trial heterogeneity and limited high-quality RCTs warrant cautious interpretation. Although platelet concentrates such as PRF or PRP are hypothesized to promote healing of extraction sockets before radiotherapy, no completed randomized controlled trials or systematic reviews currently exist to support their preventive use against ORN [33].

Given the high risk of complications, thorough dental assessment and intervention remain essential before starting head and neck radiotherapy. Pre-treatment protocols usually involve eliminating all active sources of infection—such as non-restorable teeth, abscesses, or advanced periodontal disease—together with preventive measures like topical fluoride and reinforcement of oral hygiene practices. These steps are well supported by systematic reviews and clinical studies, which consistently highlight their role in reducing oral morbidity and helping patients complete oncologic treatment without interruption [25,26,34]. In practice, patient education also plays a central role: instruction on fluoride use, oral moisturizers, and dietary modification improves adherence and lowers long-term risk. Finally, close cooperation between oncologists and dental teams allows early detection of problems and timely intervention, safeguarding both oral and general health throughout therapy [35].

## 5. Impact of Chemotherapy on Oral Health

**Figure 3:**
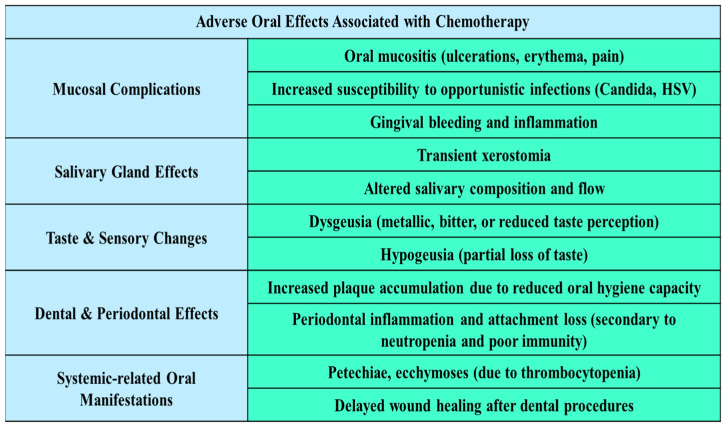
Adverse oral effects associated with chemotherapy [11,37,38].

Chemotherapy can cause a range of oral complications, many of which overlap with those observed after radiotherapy. Oral mucositis is one of the most common side effects, with a clinical presentation similar to that described in the radiotherapy section, though its onset and severity depend on the specific cytotoxic regimen. This condition is additionally exacerbated in immunocompromised patients, where even only minor lesions can also serve as entry points for systemic infections [27,36]. Xerostomia, a common side effect of chemotherapy, diminishes saliva’s protective functions and predisposes patients to increased plaque accumulation, caries, and opportunistic infections [9]. Taste alterations, often described as dysgeusia or metallic taste, further diminish appetite and quality of life [37]. Importantly, the oral complications of chemotherapy are typically transient and reversible, unlike those caused by radiotherapy [10,38]. However, they can still disrupt treatment plans if not managed proactively. Preventive dental care before initiating chemotherapy, such as the elimination of carious lesions, periodontal therapy, and oral hygiene reinforcement, is essential to reduce infection risk [39,40]. Emerging strategies offer promising adjunctive approaches to reduce oral toxicity. Low-level laser therapy (LLLT) has shown potential in reducing the severity and duration of oral mucositis by enhancing tissue regeneration and reducing inflammation; however, current evidence remains limited, and further well-designed studies are required to confirm these findings [9]. Similarly, the role of probiotic and zinc supplementation in stabilizing the oral microbiota and regenerative approaches such as stem cell therapies are under investigation for their role in maintaining mucosal integrity and enhancing healing [11,40] (Figure 3).

### Role of Pre-Chemotherapy Dental Interventions

Oral infections, whether acute or chronic, are thought to negatively impact overall health. However, acute oral infection sites like acute apical abscesses pose a greater risk if left untreated, as they can potentially spread from their initial location to other facial areas and lead to severe, life-threatening complications [41,42]. Teeth with minor decay can be treated using conventional methods, whereas teeth with irreversible pulpitis or periapical lesions require more intensive treatment such as endodontic treatment or even tooth extraction to eliminate the source of infection before beginning of chemotherapy. Ideally, the onset of chemotherapy should be delayed for at least a week after completing endodontic treatment [43]. However, recent evidence indicates that while acute oral foci should be addressed promptly, leaving chronic, asymptomatic dental issues untreated does not increase infectious complications in intensively treated patients [44,45]. In most cases, patients undergoing chemotherapy, like those with leukemia, need to begin treatment soon after diagnosis. Therefore, if oral foci of infection are detected during pre-treatment dental screening, there will not be enough time for thorough dental treatment before chemotherapy begins. Additionally, the reduced healing capacity during the untreated leukemia phase further complicates the situation. Therefore, recent studies suggest that treating acute oral infection sites (Table 1) while leaving chronic oral foci of infection (Table 1) untreated before the onset of intensive chemotherapy in patients requiring immediate chemotherapy does not increase the mortality rate related to cancer therapy [44,46]. This evolving approach may help balance the urgency of initiating chemotherapy with the need to manage oral health risks, offering a more flexible and patient-centered strategy for pre-treatment dental care [44].

## 6. Oral Health and Cardiovascular Disease

Chronic oral infection has been recognized as an independent risk factor for cardiovascular disease (CVD). Periodontal disease, in particular, has been linked to an elevated risk of infective endocarditis and the progression of atherosclerotic plaques through recurrent bacteremia, often involving pathogens such as *Porphyromonas gingivalis* and *Fusobacterium nucleatum* [47]. More recently, a systematic review and meta-analysis demonstrated that chronic apical periodontitis, frequently associated with organisms such as *Enterococcus faecalis*, may also contribute to cardiovascular risk via systemic inflammatory pathways and its relationship to atherosclerosis [48]. Consequently, dentists are advised to encourage patients to undergo routine dental clearance before cardiothoracic surgery, as this may reduce the incidence of postoperative infectious complications [49,50].

### 6.1. Profound Dental Caries and Apical Periodontitis as Surgical Risk Factors

Apical periodontitis, commonly arising from untreated deep dental caries or trauma, is characterized by inflammation and microbial infection around the root apex. Clinically, it may remain asymptomatic or present as acute pain and swelling. Standard management involves endodontic therapy to eradicate infection and preserve the tooth when feasible [15].

Beyond its local consequences, apical periodontitis contributes to systemic inflammation [51]. A systematic review and meta-analysis by Gomes et al. (2013) confirmed that patients with active lesions show elevated systemic markers, including C-reactive protein (CRP) and interleukin-1β, which decrease after successful root canal treatment [50]. More recent evidence by Al-Abdulla et al. (2023) demonstrated that effective endodontic therapy significantly lowers serum concentrations of cardiovascular disease-related biomarkers, such as high-sensitivity CRP and matrix metalloproteinases [52]. These findings reinforce that dental infections may amplify systemic inflammatory burden, which is particularly concerning in patients preparing for cardiovascular surgery.

Epidemiological studies further suggest a potential association between apical periodontitis and cardiovascular disease, although causality remains debated [53]. A 2021 meta-analysis by Koletsi et al. highlighted heterogeneity across study designs but nonetheless indicated a modest positive association between chronic endodontic infections and cardiovascular risk [49]. The proposed biological link involves dissemination of oral pathogens such as *Enterococcus faecalis* and *Fusobacterium nucleatum*, which may translocate into the bloodstream during bacteremia episodes and contribute to vascular inflammation and atherogenesis [54].

### 6.2. Necessity of Dental Evaluation and Treatment Prior to Surgery

For patients scheduled to undergo cardiovascular procedures such as valve replacement, transplantation, or ventricular assist device implantation, oral health evaluation is routinely recommended to identify and manage potential sources of infection. Active oral conditions—such as advanced caries, abscesses, or periodontitis—pose a risk of bacteremia and may exacerbate systemic inflammation, both of which can compromise postoperative recovery [51].

The strength of evidence regarding the necessity and timing of dental clearance prior to surgery, however, remains mixed. Lockhart et al. (2019) reported that oral foci of infection can contribute to postoperative complications but highlighted that most data were indirect and based on observational designs, limiting the strength of conclusions [55]. Similarly, Kouwenberg et al. (2022) emphasized that while dental screening is widely practiced, robust evidence proving that preoperative dental clearance directly prevents cardiovascular surgical complications remains limited, pointing to a need for well-designed prospective studies [56].

By contrast, more recent clinical investigations have provided supportive data. Zhou et al. (2024) analyzed outcomes in nearly 500 patients undergoing oral health clearance prior to cardiovascular surgery [57]. Their findings suggested that structured dental assessment and treatment were feasible and safe, with reduced rates of postoperative complications in patients where active oral infections were eliminated [57]. In parallel, Valizadeh et al. (2024) showed that preoperative dental intervention in cardiac surgical patients significantly decreased infectious complications and improved functional recovery, supporting the practical value of such protocols [58].

**Figure 4:**
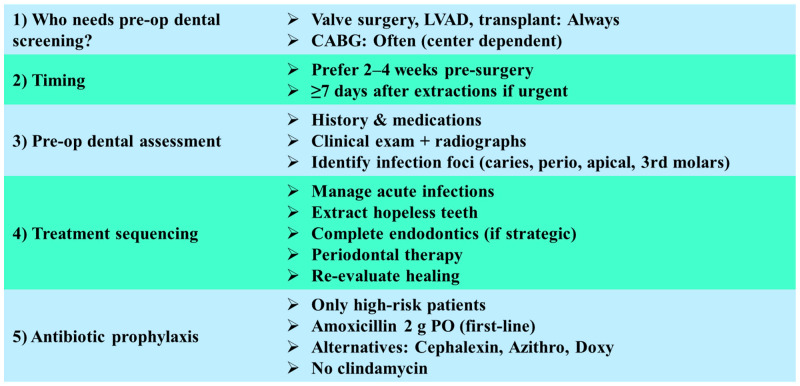
Flowchart illustrating dental management recommendations for patients scheduled for cardiovascular surgery [59,60,61,62,63].

Taken together, these studies suggest that while the evidence base is still evolving, there is growing clinical support for comprehensive preoperative dental care as a component of surgical risk reduction. A balanced approach should combine evidence-informed decision making with interdisciplinary collaboration, prioritizing acute oral infections for urgent treatment while recognizing the ongoing need for high-quality prospective trials to confirm causal benefit. In the following subsections, we outline the recommended approaches for assessing and managing dental conditions in patients preparing for cardiac surgery, with a focus on reducing postoperative risk (see Figure 4).

#### 6.2.1. Indications for Preoperative Dental Screening

Preoperative dental evaluation is considered essential for patients undergoing valve replacement or repair, left ventricular assist device (LVAD) implantation, or heart transplantation, as these individuals are at the highest risk for infective endocarditis (IE) and device-related infections. In patients scheduled for coronary artery bypass grafting (CABG) or other cardiovascular procedures, dental clearance is frequently recommended, although the evidence for improved outcomes remains mixed and may depend on institutional protocols [59,60].

#### 6.2.2. Timing of Dental Treatment

The optimal period for eliminating potential oral infection foci is two to four weeks prior to surgery, which allows for complete healing of extraction sites or periodontal interventions. Where urgent surgery is indicated, at least seven days of healing following dental extractions is advised before proceeding to cardiovascular intervention in order to reduce the risk of postoperative complications [60,61].

#### 6.2.3. Scope of Preoperative Dental Assessment

A comprehensive assessment should include a detailed medical and dental history, with emphasis on cardiovascular diagnosis, prior infective endocarditis, anticoagulant therapy, and allergy profile. Clinical and radiographic evaluation—ideally panoramic radiography supplemented with periapical or bitewing radiographs—should be performed to detect infection foci. Priority is given to identifying conditions such as symptomatic apical periodontitis, non-restorable teeth, periapical lesions, active periodontitis, pericoronitis, and impacted or symptomatic third molars [59,60].

#### 6.2.4. Sequencing of Dental Interventions

The treatment plan should follow a prioritized sequence:

1. Drainage and management of acute odontogenic infections;
2. Extraction of teeth with hopeless prognosis or non-restorable condition;
3. Completion of endodontic therapy in teeth with strategic importance and favorable prognosis;
4. Periodontal debridement and stabilization of gingival inflammation;
5. Re-evaluation of healing status prior to surgical clearance.

Although some studies suggest limited impact of mandatory preoperative dental clearance on the incidence of postoperative infective endocarditis, international guidelines continue to emphasize the elimination of oral sepsis as a critical preparatory [59,60,62].

#### 6.2.5. Antibiotic Prophylaxis in Dental Procedures

Antibiotic prophylaxis is not universally indicated for all patients undergoing preoperative dental procedures but is restricted to individuals with the highest risk cardiac conditions: those with prosthetic valves, a history of infective endocarditis, certain congenital heart diseases, and cardiac transplant recipients who develop valvulopathy. The first-line regimen is oral amoxicillin 2 g administered 30–60 min prior to the procedure. In patients with penicillin allergy, cephalexin, azithromycin, clarithromycin, or doxycycline may be considered. For those unable to take oral medications, ampicillin or cefazolin intravenously/intramuscularly is appropriate. Notably, clindamycin is no longer recommended as an alternative [59,60,63].

## 7. Conclusions

Comprehensive dental assessment and timely treatment remain critical prerequisites before the initiation of oncological therapies and major cardiovascular interventions. For clinical practice, several principles should be emphasized:

- Prioritize acute infections: Conditions such as abscesses, symptomatic apical periodontitis, and severe periodontal disease should be treated promptly, ideally at least 10–14 days before the initiation of surgery or radiotherapy, whenever the medical timeline allows;
- Tailor preventive strategies: Patients scheduled for head and neck radiotherapy should receive targeted preventive measures such as fluoride therapy, dietary counseling, and saliva preserving interventions to reduce the risk of radiation-related caries and osteoradionecrosis. For those undergoing chemotherapy, management should focus on minimizing mucositis and fungal infections, while in cardiovascular patients, preventive care should address bacteremia risk and the potential for infective endocarditis;
- Balance urgency and feasibility: In urgent oncological or cardiac cases, clinicians may need to address acute infections rapidly while deferring management of chronic, asymptomatic conditions. This highlights the importance of interdisciplinary communication between dental, oncology, and cardiology teams;
- Integrate new tools and evidence: Emerging approaches such as adjunctive platelet-rich fibrin (PRF) in extraction sites, advanced imaging for detecting subclinical oral infections, and structured patient education protocols may support more precise, risk-based dental decision making.

Looking forward, high-quality prospective studies are essential to establish the optimal timing and extent of dental interventions in medically compromised patients. However, current evidence supports the integration of systematic dental clearance into oncological and cardiac care pathways as a practical strategy to lower postoperative and treatment-related complications

## Figures and Tables

**Table 1:** The following oral diseases should be considered as acute or chronic foci of infection [44].

Chronic Oral Foci of Infection	Acute Oral Foci of Infection
- Periodontal pockets ≥ 6 mm - Periapical granuloma - Initial endodontic treatment - Furcation involvement - Retained roots - Fully or partially impacted teeth - Caries profunda - Follicular cyst	- Active pus-producing fistula - Symptomatic periapical granuloma

## Data Availability

Data supporting our results are available for request from the corresponding author.
